# PPP2CA Is a Novel Therapeutic Target in Neuroblastoma Cells That Can Be Activated by the SET Inhibitor OP449

**DOI:** 10.3389/fonc.2022.744984

**Published:** 2022-06-22

**Authors:** Celimene Galiger, Meike Dahlhaus, Michael Peter Vitek, Klaus-Michael Debatin, Christian Beltinger

**Affiliations:** ^1^ Section of Experimental Pediatric Oncology, Department of Pediatrics and Adolescent Medicine, University Medical Center, Ulm, Germany; ^2^ Cognosci, Inc., Research Triangle Park, NC, United States; ^3^ Department of Neurology, Duke University Medical Center, Durham, NC, United States; ^4^ Department of Pediatrics and Adolescent Medicine, University Medical Center Ulm, Ulm, Germany

**Keywords:** neuroblastoma, PPP2CA, SET inhibitor, OP449, dasatinib, AKT

## Abstract

Neuroblastoma (NB) is the most common extracranial solid tumor in childhood and has a poor prognosis in high-risk cases, requiring novel therapies. Pathways that depend on phospho-signaling maintain the aggressiveness of NB. Protein phosphatase 2 (PP2A) with its catalytic subunit PPP2CA is a major phosphatase in cancer cells, including NB. We show that reduction of PPP2CA by knock-down decreased growth of NB cells and that complete ablation of PPP2CA by knock-out was not tolerated. Thus, NB cells are addicted to PPP2CA, an addiction augmented by *MYCN* activation. SET, a crucial endogenous inhibitor of PP2A, was overexpressed in poor-prognosis NB. The SET inhibitor OP449 effectively decreased the viability of NB cells, independent of their molecular alterations and in line with a tumor suppressor function of PPP2CA. The contrasting concentration-dependent functions of PPP2CA as an essential survival gene at low expression levels and a tumor suppressor at high levels are reminiscent of other genes showing this so-called Goldilocks phenomenon. PP2A reactivated by OP449 decreased activating phosphorylation of serine/threonine residues in the AKT pathway. Conversely, induced activation of AKT led to partial rescue of OP449-mediated viability inhibition. Dasatinib, a kinase inhibitor used in relapsed/refractory NB, and OP449 synergized, decreasing activating AKT phosphorylations. In summary, concomitantly reactivating phosphatases and inhibiting kinases with a combination of OP449 and dasatinib are promising novel therapeutic approaches to NB.

## Introduction

Neuroblastoma (NB) is the most common extracranial solid tumor in childhood [reviewed in (1, 2)]. Alterations in copy number; amplification of *MYCN* or expression of MYC; mutations of *ALK*, *PTPN11*, *ATRX*, *LMO1*, and RAS-RAF-MAPK pathway genes; genomic alterations and overexpression of *LIN28B*; and genomic rearrangements and alternative mechanisms of activating *TERT* are important in the pathogenesis of NB (3–15). Many of these genetic drivers in NB mediate their oncogenic action by phosphorylation reactions within the MYCN, MYC, ALK, RAS-RAF-MAPK, and AKT signaling pathways, which can also be activated by non-genetic mechanisms in NB. Phosphorylation also determines the efficacy of antiapoptotic and proapoptotic proteins, which are frequently dysfunctional in NB, including BCL2, BAD, BIM, and AURKA. Despite the advances in understanding NB, the prognosis of high-risk patients is still poor. Thus, better drugs are urgently needed for this patient group.

**Figure 1:**
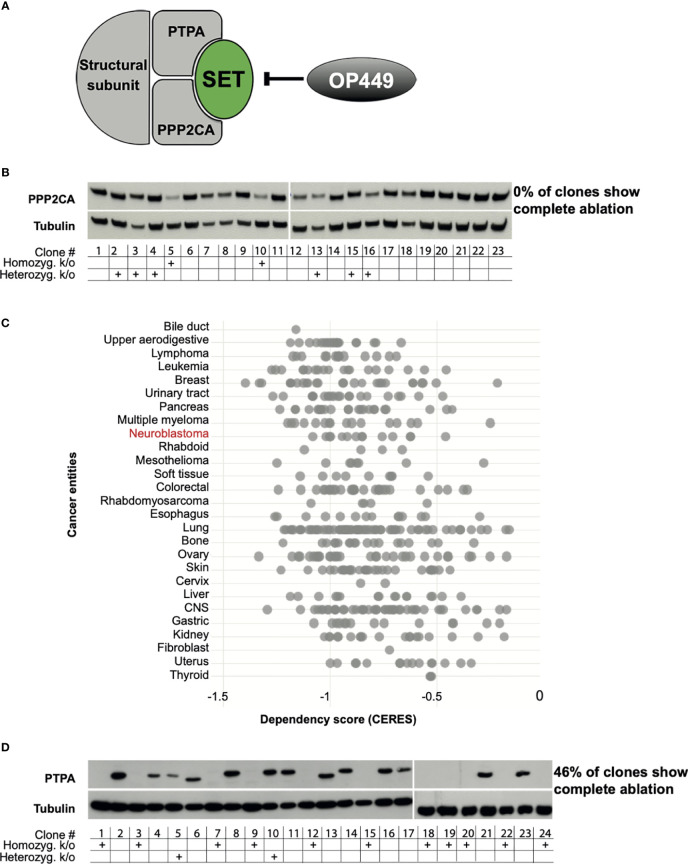
PPP2CA, but not PTPA, is an essential survival gene for neuroblastoma (NB) cells. **(A)** Structure of PP2A with the subunits PPP2CA and PTPA, the PP2A inhibitor SET, and the SET inhibitor OP449. **(B)** KELLY NB cells do not tolerate complete lack of PPP2CA. Cells were transfected with a vector expressing guiding RNA against PPP2CA and expressing Cas9. Single cell-derived clones were analyzed. Expression of PPP2CA was assessed by Western blotting, with tubulin as loading control. The presence of the *PPP2CA* alleles, or its homozygous or heterozygous knock-out, was determined by Sanger sequencing. **(C)** Additional NB cell lines do not tolerate complete lack of PPP2CA*. In silico* analysis of genome-scale CRISPR/Cas9 knock-out screens of cancer cell lines including NB using the DepMap database (https://depmap.org/portal/) (18–20). A negative CERES score indicates that a gene is essential for survival. **(D)** KELLY NB cells tolerate complete lack of PTPA. Cells were transfected with a vector expressing guiding RNA against PTPA and expressing Cas9. Expression of PTPA in single clones was assessed by Western blotting, with tubulin as control. The presence of the *PTPA* alleles, or its homozygous or heterozygous knock-out, was determined by Sanger sequencing.

Protein phosphatase 2 (PP2A) is one of the four important serine/threonine phosphatases in mammalian cells (16) and an important tumor suppressor in many cancer types, where it is frequently downregulated or post-translationally modified [reviewed in (17)]. The holoenzyme consists of three different subunits: catalytic, structural, and regulatory subunits (**Figure 1A**). Each subunit exists in isoforms: two catalytic, two structural, and twelve regulatory subunits. PPP2CA, the alpha isoform, is the major catalytic subunit, and PTPA is an activating regulatory subunit. A pivotal endogenous inhibitor of PP2A is SET (21), which has thus become an attractive target for pharmacological reactivation of PP2A [reviewed in (22)].

Among other growth-promoting targets, PP2A inhibits the RAS-RAF-MAPK pathway in various cancers [reviewed in (17) and (22)]. In addition, PP2A induces apoptosis by dephosphorylating and thus inactivating several antiapoptotic proteins, including phospho-AKT (17). As noted above, these mitosis- and apoptosis-related proteins play a crucial role in the growth of NB. However, little is known about the tumor-suppressive role of PP2A in NB (23).

Given the importance of PP2A in reversing oncogenic phosphorylation and since SET is a crucial endogenous inhibitor of PP2A, the expression of SET in cancers and its pharmacological reactivation have been the focus of intense investigations. Thus, SET has been shown to be overexpressed in a multitude of cancers (24–29).

Compared with several other PP2A-reactivating drugs (PADs) (24, 30–32), the novel SET antagonist OP449 has been shown to be particularly effective and devoid of side effects on normal cells. OP449 is a cell-penetrating peptide able to interact with the SET oncoprotein, thus antagonizing SET’s inhibition of PP2A (33, 34). With the use of cell lines and patient-derived cells, OP449 has been shown to increase PP2A activity and to control B-cell NHL (24), CLL (24), T-ALL (25), CML (26), AML (26), prostate cancer (27), pancreatic cancer (28), breast cancer (29, 35), and oral squamous cell cancer (36). Growth control by OP449 of these diverse tumor entities was associated with dephosphorylation of crucial signaling molecules, of which AKT and ERK also determine tumor aggressiveness of NB. However, it is yet unknown whether OP449 has such effects in NB. We show in this paper that PPP2CA is a novel therapeutic target in NB cells that can be activated by the SET inhibitor OP449.

## Materials and Methods

### Reagents

4-Hydroxytamoxifen (59002) was obtained from Merck Chemicals (Darmstadt, Germany), doxycycline (631311) from Clontech Laboratories (Mountain View, CA, USA), and PP2A Immunoprecipitation Phosphatase Assay Kit (17-313) from Merck Millipore (Darmstadt, Germany). Dasatinib (S1021), dactolisib (S1009), sorafenib (S7397), erlotinib (S7786), lorlatinib (S7536), afatinib (S1011), trametinib (S2673), and SC79 (S7863) were from Selleck (Munich, Germany). OP449 was provided by Oncotide Pharmaceuticals (Durham, NC, USA).

### Cell Culture

The human NB cell lines SK-N-AS, SK-N-SH, and SK-N-BE(2)-C were purchased from ATCC (LGC Standard, Wesel, Germany). GI-M-EN, SH-SY5Y, IMR32, LAN5, and KELLY cells were from DSMZ (Braunschweig, Germany), and NB69 cells were from ECACC (Salisbury, UK). IMR5 and NLF cells were a gift from G. M. Brodeur (CHOP, Philadelphia, PA, USA). SK-N-AS and SK-N-SH cells were cultured in Dulbecco’s modified Eagle’s medium (DMEM; Gibco, Paisley, UK) supplemented with 10% of heat-inactivated fetal calf serum (FCS; Gibco), SH-SY5Y cells in DMEM with 20% FCS, and SK-N-BE(2)-C cells in a 1:1 mixture of EMEM and Ham’s F12 (Gibco) with 10% FCS. GI-M-EN, SH-EP, NLF, and KELLY cells were grown in Roswell Park Memorial Institute (RPMI) 1640 medium (Gibco) with 10% FCS, NB69 in RPMI 1640 medium with 15% FCS, and IMR5, IMR32, and LAN5 cells in RPMI 1640 medium with 20% FCS. All media were supplemented with 2 mM of l-glutamine (Gibco) and 100 U/ml of penicillin/streptomycin (Gibco). The authenticity of cells was verified by short tandem repeat (STR) profiling using the Cell Line Authentication Kit (Sigma-Aldrich, Taufkirchen, Germany). Cells were tested for mycoplasma contamination using the LookOut Mycoplasma PCR Detection Kit (MP0035, Sigma-Aldrich).

### Knock-Out of PPP2CA and PTPA

GeneArt CRISPR Nuclease Vectors (Life Technologies, Darmstadt, Germany) expressing guiding RNA against PPP2CA or PTPA, and Cas9 and GFP were lipofected into KELLY NB cells. Transfected cells were sorted for green fluorescent protein (GFP) expression and deposited as single cells in 96-well plates. Clones from single cells were randomly picked and subjected to Western blotting for PPP2CA, PTPA, and control tubulin. The percentage of clones with complete ablation of PPP2CA and PTPA proteins, of all clones investigated, was calculated. Sanger sequencing was used to determine the presence of premature stop codons leading to homozygous or heterozygous gene knock-outs.

### MYCN Activation, RNA Isolation, and Quantitative Real-Time PCR

SH-EP MYCN-ER cells were used as previously described (37). Cells were cultured in the presence or absence of 300 nM of 4-hydroxytamoxifen (4-OHT). Total RNA was extracted using Direct-zol RNA Kit (R2051, Zymo Research, Freiburg, Germany) according to the manufacturer’s instructions and reverse-transcribed using SuperScipt III (18080-51, Invitrogen, Darmstadt, Germany). Gene expression of *ODC1* was determined by quantitative real-time PCR 48 h after activation of MYCN using forward primer GCATGTGGTGATTGGATGGTGTT, reverse primer TGGCTCTGGATCTGCCTTCATGAGT, and Advanced Universal SYBER Green Supermix (1725274, Bio-Rad, Feldkirchen, Germany) in a CFX Connect Thermal Cycler (Bio-Rad).

### Knock-Down of PPP2CA

SH-EP MYCN-ER cells were stably transduced with two lentiviral shRNAs targeting PPP2CA or a control shRNA with a non-silencing sequence at a multiplicity of infection (MOI) of 50 (TRIPZ, RHS4696-201904073, RHS4696-201904413, and RHS5087-EG332; Dharmacon, CO, USA) according to the manufacturer’s instructions. After selection with puromycin, cells were treated with 5 μg/ml of doxycycline to induce the expression of shRNA. Knock-down efficiency of PPP2CA was assessed by Western blotting.

### Cell Growth and Apoptosis Assays

SH-SY5Y and KELLY cells were seeded at 5 × 10^4^ cells per well or SH-EP *MYCN*-ER cells were seeded at 2 × 10^4^ cells per well into 6-well plates overnight. SH-SY5Y and KELLY cells were treated with increasing concentrations of OP449 on day 1. With the use of flow cytometry, dead, i.e., propidium iodide-positive, cells were excluded, and viable cells were counted. SH-EP *MYCN*-ER cells were treated with or without 4-OHT, and shRNA was induced by 1 µg/ml of doxycycline. Apoptosis was determined by quantification of DNA fragmentation using fluorescence-activated cell sorting (FACS) analysis of propidium iodide-stained nuclei. Cells were resuspended in 300 µl of hypotonic fluorochrome solution containing 0.1% sodium citrate, 0.1% Triton X-100, and 50 µg/ml of propidium iodide in distilled water. Cells were incubated at 4°C overnight, and hypodiploid nuclei were determined by flow cytometry. Specific apoptosis was calculated according to the following formula: 100 × [experimental cell death (%) − spontaneous cell death (%)]/[100 − spontaneous cell death (%)].

### Clonogenic Growth Assay

In supplemented RPMI 1640 medium for KELLY and SH-EP *MYCN*-ER cells and supplemented DMEM for SH-SY5Y cells, 0.75 × 10^3^ cells per well were seeded in triplicates into 6-well plates and allowed to attach overnight. SH-SY5Y and KELLY were treated with OP449 on days 1 and 3. SH-EP *MYCN*-ER cells were treated with vehicle or with 4-OHT, and shRNA was induced by 1 µg/ml of doxycycline. After 1 week (SH-EP *MYCN*-ER cells) and 2 weeks (SH-SY5Y and KELLY cells) in culture, colonies were stained with crystal violet solution in 3.7% formaldehyde.

### Soft Agar Growth Assay

Experiments were carried out in triplicates in 24-well plates coated with a layer of 0.6% low-gelling-temperature agarose (A0701, Sigma-Aldrich) with DMEM or RPMI medium. In 0.5% agar with DMEM or RPMI medium, 2 × 10^3^ cells per well were seeded as a single-cell suspension. OP449 in 1 ml of culture medium was added above the top agar on days 1 and 3 in SH-SY5Y and KELLY cells. For the SH-EP *MYCN*-ER cell soft agar assay, the medium contained 300 nM of 4-OHT and 1 µg/ml of doxycycline in 1 ml of culture medium, which was exchanged every 48 h. After 1 week (SH-EP *MYCN*-ER cells) and 2 weeks (SH-SY5Y and KELLY cells) in culture, colonies that had formed within the soft agar were stained with 1 mg/ml of MTT in phosphate-buffered saline (PBS).

### OP449 Viability Assay

Cells measuring 1 × 10^4^ were plated in 96-well plates, incubated overnight, and then exposed for 24, 48, 72, and 96 h to increasing concentrations of OP449. Cell viability was determined by MTT assay, with the viability of dimethyl sulfoxide (DMSO)-treated controls set at 100%.

### SC79 Plus OP449 Viability Assay

Cells measuring 3 × 10^4^ were plated in 96-well plates and incubated overnight. Cells were pretreated for 0.5 h with SC79 at 10 µM for KELLY cells and 20 µM for SH-SY5Y cells. SC79 was continued for an additional 2 h in the presence of increasing doses of OP449. Cell viability was determined by MTT assay, with the viability of DMSO- or PBS-treated controls set at 100%.

### *In Silico* Analysis of Neuroblastoma Patients

The R2: Genomics and Visualization Platform (http://r2.amc.nl) was used to analyze the expression of SET mRNA (gse45545) and SET protein (38) in NB patients.

### PP2A Activity

PP2A activity of SH-SY5Y and KELLY cells was determined using PP2A Immunoprecipitation Phosphatase Assay Kit (17-313, Merck) according to the manufacturer’s protocol. Cells were treated with increasing concentrations of OP449 for 4 h and lysed, and PPP2CA, the catalytic subunit of PP2A, was immunoprecipitated. The precipitate was incubated with K-R-pT-I-R-R as a substrate and with Malachite Green for the detection of released (free) phosphate. PP2A activity was measured in supernatants at 450 nm using a TECAN microplate reader (TECAN, Männedorf, Switzerland).

### Western Blotting Analysis

Cells were lysed in radioimmunoprecipitation assay (RIPA) buffer (Tris pH 8, 50 mM), NaCl (150 mM), 1% NP-40, 0.1% sodium dodecyl sulfate (SDS), 1% sodium deoxycholate (DOC), EDTA (pH 8, 1 mM), 2 mM of dithiothreitol (DTT), and Protease Inhibitor Cocktail EDTA-free (Roche, Basel, Switzerland). Whole protein lysate measuring 20 µg was resolved on 4%–12% Bis-Tris gels (NP0335BOX, P0323BOX, Invitrogen). Nitrocellulose membranes were blocked with 5% non-fat dry milk in Tris-buffered saline with Tween 20 (25 mM of Tris-HCl (pH 7.4), 137 mM of NaCl, and 0.1% Tween 20) and then incubated overnight at 4°C with the following antibodies: mouse anti-phospho-PPP2CA (sc-271903, Santa Cruz Biotechnology, Heidelberg, Germany, 1:500) and mouse anti-PPP2CA (610555, BD Transduction Laboratories, CA, USA, 1:5,000). For the detection of phosphorylated proteins, cells were lysed in RIPA buffer (Tris-HCl pH 7.5, 10 mM), NaCl (150 mM), EDTA (2 mM), 1% Triton-X 100, 10% glycerol, and DTT (2 mM), and Western blotting analyses were performed with rabbit anti-ERK1/2 (M5670, Sigma, 1:10,000), mouse anti-ERK-phospho-Thr202/Tyr204 (612358 BD Transduction Laboratories, 1:2,000), rabbit anti-AKT (9272, Cell Signaling Technologies, Frankfurt, Germany, 1:1,000), mouse anti-AKT-phospho-Thr308 (558316 BD Transduction Laboratories, 1:2,000), rabbit anti-AKT-phospho-Ser473 (9271, Cell Signaling Technologies, 1:2,000), rabbit anti-p70S6-phospho-Thr389 (9205, Cell Signaling Technologies, 1:1,000), and p70S6 (9202, Cell Signaling Technologies, 1:1,000) antibodies.

As secondary antibodies, goat anti-mouse IgG (H+L)–horseradish peroxidase (HRP) conjugate (170-6516, Bio-Rad, 1:10,000) and goat anti-rabbit IgG (H+L)-HRP conjugate (65-6120, Invitrogen, 1:10,000) were used for 1 h at room temperature.

For detection, enhanced chemiluminescence (ECL) (GE Healthcare, Buckinghamshire, UK) and WESTAR ETA ULTRA (Cyanagen, Bologna, Italy) reagents were employed.

### Drug Combination Analysis

The concentration ranges of OP449 and kinase inhibitors were selected to span concentrations below and above the IC_50_ values for each drug. The ratio of the drugs in combinations was fixed according to the ratio of the IC_50_ of the drugs. The combination index (CI) of survival rates in the combination studies was calculated according to the Chou–Talalay method (39) using CompuSyn software. Weighted average CI values were calculated as CI_wt_ = (CI_50_ + 2CI_75_ + 3CI_90_ + 4CI_95_)/10, where CI_50_, CI_75_, CI_90_, and CI_95_ represent the CI values obtained at 50%, 75%, 90%, and 95% inhibition of cell viability, respectively (39).

## Results

### PPP2CA Is an Essential Survival Gene in Neuroblastoma Cells

To assess whether PPP2CA may be amenable to therapeutic inhibition, we ablated its gene in NB cells using CRISPR/Cas9. The KELLY cell line was selected, as this is a paradigmatic *MYCN*-amplified NB cell line with mutated ALK, p53, and ARF. In addition, the copy number of 9q34 is increased, where both PTPA, the regulatory subunit of PP2A that activates PPP2CA, and SET, the major endogenous inhibitor of PP2A, are located. We reasoned that if PPP2CA were essential for NB viability, then few, if any, knock-out clones lacking PPP2CA should be generated, as such clones would die out after knock-out. Indeed, none of the many randomly picked clones that grew out had lost PPP2CA protein (**Figure 1B**). The lack of homozygous knock-out in the surviving clones was confirmed on the genomic level by sequencing (**Figure 1B**). Only two clones had a homozygous knock-out, and these two clones still harbored PPP2C protein, possibly an alternative PPP2C isoform. Of note, in the heterozygous knock-outs, the remaining allele apparently upregulated PPP2CA protein expression, as protein levels were not decreased (**Figure 1B**). These data show that KELLY NB cells do not tolerate a complete lack of PPP2CA. *In silico* data mining of genome-scale CRISPR/Cas9 knock-out screens confirmed the dependency of KELLY cells on PPP2CA and extended this conclusion to other NB cell lines and additional cancer entities (**Figure 1C**). In contrast to ablation of PPP2CA, knock-out of PTPA in KELLY cells resulted in the outgrowth of many clones with a homozygous knock-out (46% of randomly picked clones), all of them completely devoid of PTPA protein (**Figure 1D**). Thus, these data strongly suggest that PPP2CA, but not PTPA, is an essential survival gene in NB and thus a suitable target for its therapy.

### PPP2CA Depletion Decreases Survival of SH-EP MYCN-ER Neuroblastoma Cells, Augmented by *MYCN* Activation

**Figure 2:**
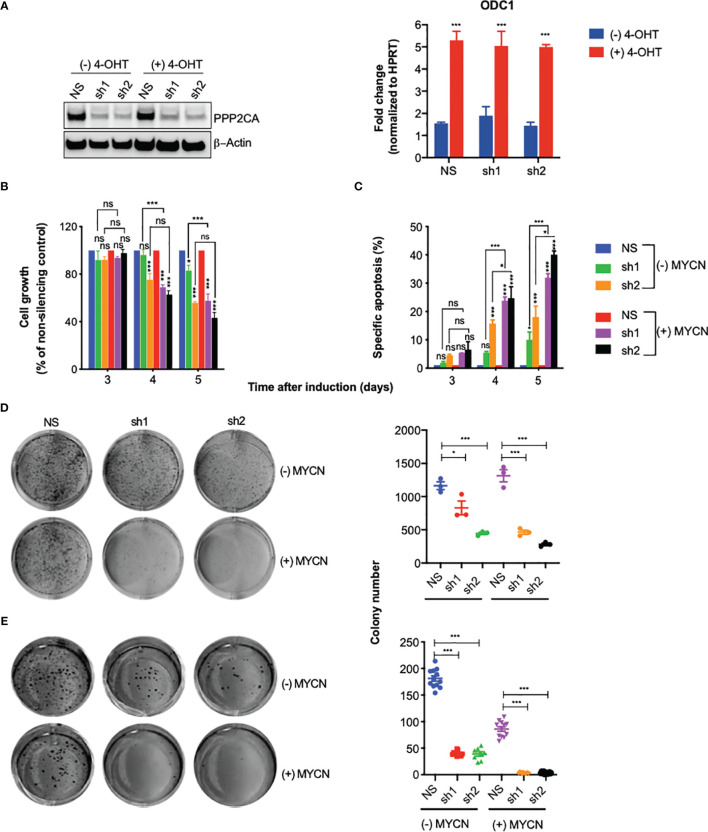
Knock-down of PPP2CA inhibits aggressiveness of SH-EP-MYCN-ER neuroblastoma (NB) cells, enhanced by MYCN activation. SH-EP-MYCN-ER cells with (+) or without (−) activation of MYCN by 4-hydroxytamoxifen (4-OHT) were treated with doxycycline to induce expression of lentivirally transduced shRNAs. NS, non-silencing control; sh1, PPP2CA-silencing shRNA1; sh2, PPP2CA-silencing shRNA2. **(A)** Knock-down of PPP2CA and activation of MYCN. Western blotting of PPP2CA and β-actin (left panel). Full-length Western blotting is shown in **Supplementary Figure 2A**. qRT-PCR of ODC1 normalized to HPRT (right panel). **(B)** Inhibition of cell growth. Viable, i.e., Hoechst stain-negative, cells were enumerated by flow cytometry. Cell growth as percentage of cells compared to non-silencing control is shown for different time points after induction by doxycycline. **(C)** Induction of apoptosis. Apoptosis was determined by enumerating hypodiploid propidium iodide-stained nuclei using flow cytometry. Shown is the specific percentage of apoptotic nuclei. **(D)** Inhibition of clonogenicity. Cells were grown in plastic dishes for 1 week and stained with crystal violet, and colonies were counted. **(E)** Inhibition of anchorage-independent growth. Cells were grown in soft agar for 1 week and stained with MTT, and colonies were counted. Experiments were repeated three times. Data are expressed as means ± SEM of triplicates and analyzed by two-way ANOVA test. *p < 0.05, ***p < 0.001; ns, not significant.

Being unlikely that therapeutic targeting of PPP2CA can achieve complete inactivation of PPP2CA, we investigated the effects of incomplete depletion of PPP2CA by knocking down PPP2CA in SH-EP MYCN-ER NB cells. As we wanted to know how MYCN influences the effects of PP2A inhibition, SH-EP MYCN-ER cells, where MYCN can be activated by the addition of 4-hydroxytamoxifen (4-OHT), were used. Induction of PPP2CA lentiviral shRNAs in SH-EP MYCN-ER repressed expression of PPP2CA in MYCN-activated and non-activated cells (**Figure 2A**, left panel). MYCN activation was verified by induction of *ODC1*, a *bona fide* transcriptional target gene of MYCN (**Figure 2A**, right panel).

Knock-down of PPP2CA decreased cell growth (**Figure 2B**), increased apoptosis (**Figure 2C**), and decreased clonogenicity (**Figure 2D**) and anchorage-independent growth (**Figure 2E**). All these effects were more pronounced when MYCN was activated (**Figures 2B–E**). Together, these results show that depletion of PPP2CA decreases the survival of NB cells, augmented by MYCN.

### SET Is Overexpressed in Poor-Prognosis Neuroblastoma

**Figure 3:**
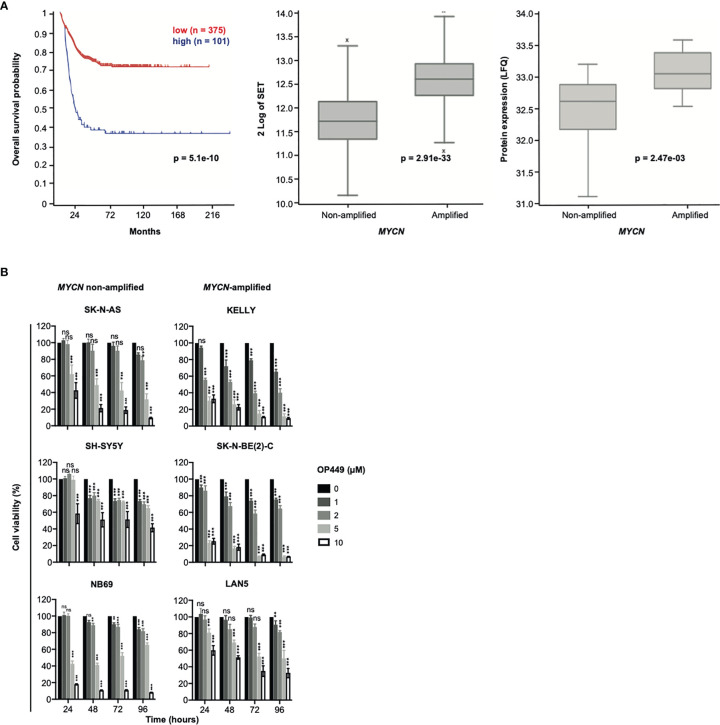
SET is overexpressed in poor-prognosis neuroblastoma (NB), and the SET inhibitor OP449 inhibits NB cells. **(A)** SET overexpression in poor-prognosis NB. Overall Kaplan–Meier survival estimates of 649 NB patients (gse45545) depending on SET transcript levels, as determined by microarray analysis and dichotomized by maximally selected log-rank statistics, are shown in the left panel. SET mRNA expression in *MYCN*-amplified and non-amplified tumors of the same patient cohort is depicted in the middle panel and SET protein expression of a different patient cohort (38) in the right panel. **(B)** OP449 decreases viability of NB cells. NB cell lines were treated with OP449 at increasing doses and duration. Cell viability was determined by MTT assay and normalized to untreated controls. Experiments were repeated three times. Data are expressed as means ± SEM of triplicates and analyzed by two-way ANOVA test. *p < 0.05, **p < 0.01, ***p < 0.001; ns, not significant.

As the role of SET in NB patients is unknown, we investigated the association of SET expression with patient survival and *MYCN* copy number in a large patient cohort. High mRNA expression of SET was associated with markedly decreased overall survival (**Figure 3A**, left panel) and with amplification of *MYCN* (**Figure 3A**, middle panel). Along this line, high protein expression of SET was associated with *MYCN* amplification in a different patient cohort (**Figure 3A**, right panel). Thus, SET is overexpressed in poor-prognosis NB patients.

### The SET Inhibitor OP449 Effectively Decreases Viability of Neuroblastoma Cells Independent of Their Molecular Alterations

Given the strong expression of SET in poor-prognosis NB, including *MYCN*-amplified NB, we asked whether inhibition of SET by OP449 would decrease the viability of NB cells. NB cell lines with non-amplified *MYCN* (SK-N-AS, SH-SY5Y, NB69), amplified *MYCN* (KELLY, SK-N-BE(2)-C, LAN5), 1p36 deletion (NB69, SK-N-AS, SK-N-BE(2)-C), activating ALK mutations (SH-SY5Y, KELLY, LAN5), inactivating p53 mutations (SK-N-AS, KELLY, SK-N-BE(2)-C), and ARF deletions (SK-N-AS, KELLY) were treated with OP449 (**Figure 3B**). In these NB cells with diverse molecular alterations, OP449 markedly decreased viability, independent of their underlying molecular alterations (**Figure 3B**).

### OP449 Decreases Aggressiveness of Neuroblastoma Cells

**Figure 4:**
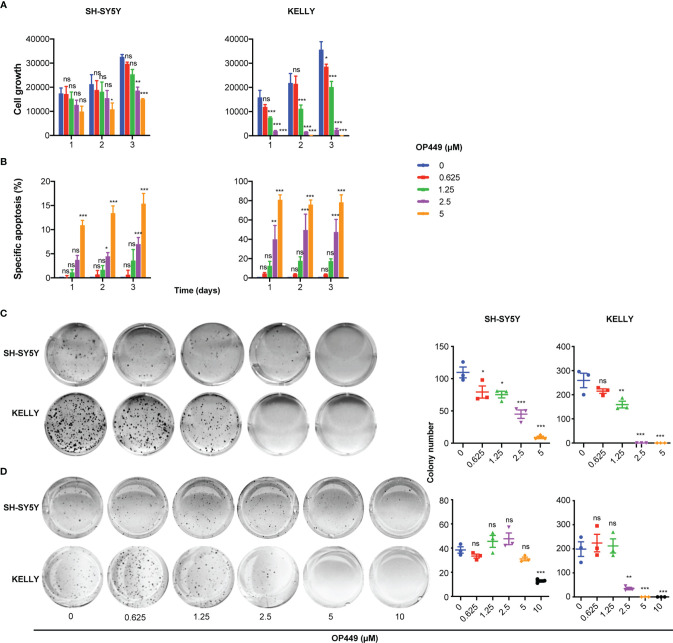
OP449 inhibits aggressiveness of SH-SY5Y and KELLY cells. *MYCN*-non amplified SH-SY5Y and *MYCN*-amplified KELLY cells were treated with increasing concentrations of OP449. **(A)** Inhibition of cell growth. With the use of flow cytometry dead, i.e., propidium iodide-positive, cells were excluded, and viable cells were counted. Shown is the number of living cells counted at different time points after start of treatment. **(B)** Inhibition of apoptosis. Apoptosis was determined by enumerating hypodiploid propidium iodide-stained nuclei using flow cytometry. Shown is the specific percentage of apoptotic nuclei. **(C)** Inhibition of clonogenicity. Cells were grown in plastic dishes and treated on days 1 and 3 after seeding. After 2 weeks, cells were stained with crystal violet, and colonies were counted. **(D)** Inhibition of anchorage-independent growth. Cells were grown in soft agar and treated on days 1 and 3 after seeding. After 2 weeks, cells were stained with crystal violet, and colonies were counted. Experiments were repeated three times. Data are expressed as means ± SEM of the means of triplicates of each independent experiment and are analyzed by two-way ANOVA test. *p < 0.05, **p < 0.01, ***p < 0.001; ns, not significant.

Next, we investigated the effects of OP449 in NB cells in more detail in the paradigmatic *MYCN* non-amplified SH-SY5Y and *MYCN*-amplified KELLY cells. OP449 attenuated cell growth, induced apoptosis, and inhibited clonogenicity in a dose-dependent manner (**Figures 4A–C**). Anchorage-independent growth was inhibited at higher doses (**Figure 4D**). Taken together, OP449 decreases the aggressiveness of SH-SY5Y cells and, more pronounced, of the *MYCN-*amplified KELLY NB cells.

### OP449 Reactivates PP2A in Neuroblastoma Cells

**Figure 5:**
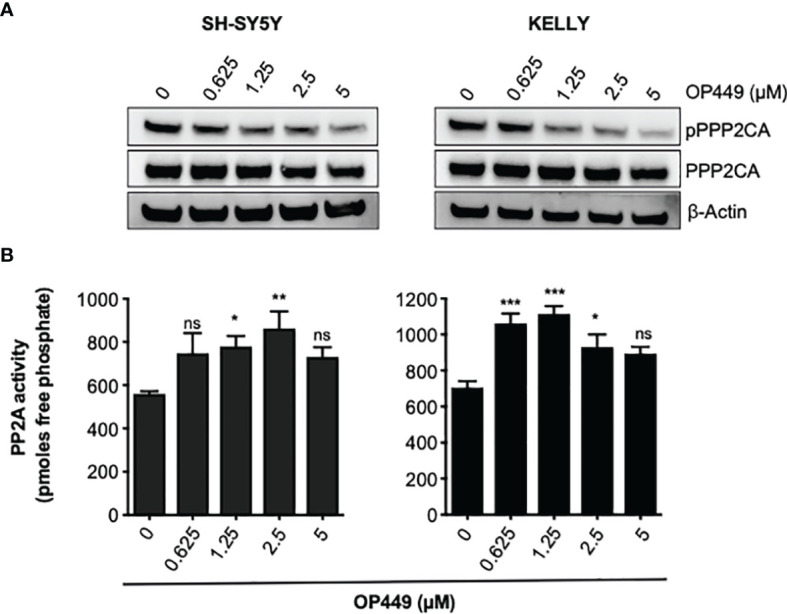
OP449 reactivates PP2A in neuroblastoma (NB) cells. **(A)** OP449 dephosphorylates PPP2CA at Y307. SH-SY5Y and KELLY NB cells were treated for 4 h with increasing concentrations of OP449. Western blotting for pY307 of PPP2CA and for PPP2CA is shown. β-Actin was used as loading control. Full-length Western blotting is shown in **Supplementary Figure 2B**. **(B)** OP449 reactivates PP2A. Cells were treated for 4 h with increasing concentrations of OP449 and lysed. PPP2CA was immuno-precipitated, and PP2A activity was determined by colorimetric assay. Experiments were repeated three times. Data are expressed as means ± SEM of triplicates and are analyzed by one-way ANOVA test. *p < 0.05, **p < 0.01, ***p < 0.001; ns, not significant.

Phosphorylation of Y307 inhibits PPP2CA (40). Treatment of SH-SY5Y and KELLY with OP449 decreased phosphorylation of PPP2CA at Y307 (**Figure 5A**), suggesting activation of PP2A. Determination of the enzymatic activity of PP2A proved reactivation of PP2A by OP449 in NB cells (**Figure 5B**).

### OP449 and Kinase Inhibitors Synergize in SH-SY5Y and KELLY Neuroblastoma Cells

**Figure 6:**
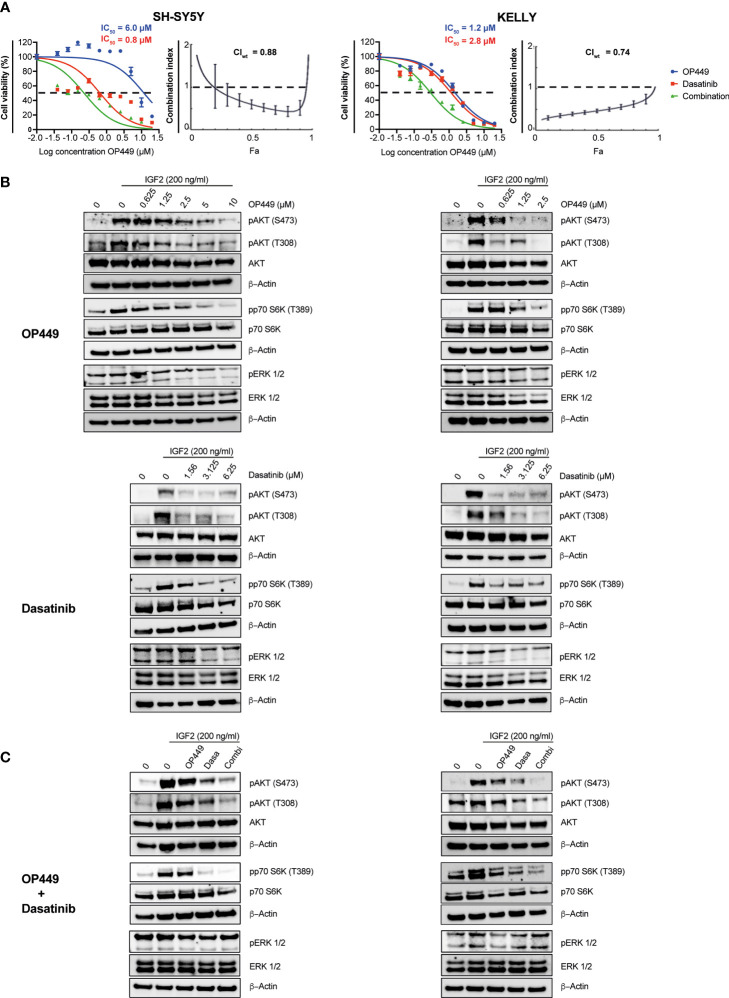
Synergy of OP449 and dasatinib in SH-SY5Y and KELLY neuroblastoma (NB) cells. **(A)** OP449 and dasatinib synergistically decrease viability. Cells were treated for 72 h with OP449, dasatinib, or a combination of both. Cell viability was determined by MTT assay and is depicted as a percentage of untreated cells, with IC_50_ values being shown. Dose–response curves for SH-SY5Y cells are plotted as log of OP449 concentrations ranging from 0 to 20 µM at a molar ratio of 1 (OP449) to 4 (dasatinib) and for KELLY cells as log of OP449 concentrations from 0 to 20 µM at a molar ratio of 1 (OP449) to 2.5 (dasatinib). Combination index (CI) and weighted average combination index (CI_wt_) were determined according to the Chou–Talalay method. Fa (fraction affected)–CI plots are shown, and the CI_wt_ is stated. A CI or CI_wt_ of less than 1 indicates synergy, 1 an additive effect, and greater than 1 an antagonist effect. The vertical bars on Fa-CI plots represent 95% confidence intervals based on sequential deletion analysis (SDA) using CompuSyn (41). Experiments were repeated three times. **(B)** OP449 and dasatinib inhibit activating phosphorylation of AKT and p70S6K. Cells were treated with increasing doses of OP449 for 4 h (top panels) or of dasatinib for 2 h (bottom panels) before being stimulated with IGF2 for 30 min. Western blotting of total lysates is shown. β-Actin was used as loading control. Full-length Western blotting is shown in **Supplementary Figures 3–6**. **(C)** OP449 and dasatinib synergize in decreasing activating phosphorylation of AKT and p70S6K. SH-SY5Y cells were treated with 1.25 µM of OP449 for 4 h, 3.125 µM of dasatinib (Dasa) for 2 h, or a combination (Combi) of both drugs. KELLY cells were treated with 2.5 µM of OP449 for 4 h, 6.25 µM of dasatinib (Dasa) for 2 h, or a combination (Combi) of both drugs. Cells were then stimulated with 200 ng/ml of IGF2, and their lysates were analyzed by Western blotting. Full-length Western blotting is shown in **Supplementary Figures 7, 8**.

We reasoned that the SET inhibitor OP449 may synergize with rationally chosen kinase inhibitors known to be efficacious in NB, as both act on phospho-signaling pathways important for the maintenance of NB. The efficacy of OP449 and the kinase inhibitors dasatinib, dactolisib, sorafenib, erlotinib, lorlatinib, afatinib, and trametinib was determined in SH-SY5Y and KELLY cells. These cell lines were differentially sensitive to OP449 (**Figure 6A** and **Supplementary Figure 1**). The various kinase inhibitors differed in their IC_50_ within and between the two cell lines (**Figure 6A** and **Supplementary Figure 1**). The synergy between OP449 and the kinase inhibitors was evaluated. As determined by the weighted average CI (CI_wt_), which emphasizes the more relevant higher inhibitory efficacies, OP449 synergized with dasatinib and dactolisib in SH-SY5Y cells and with dasatinib, sorafenib, erlotinib, and lorlatinib in KELLY cells (**Figure 6A** and **Supplementary Figure 1**). Thus, dasatinib, but not the other kinase inhibitors, exhibited relevant synergy with OP449 in both cell lines.

### OP449 and Dasatinib Synergistically Inhibit Activating Phosphorylations in the AKT Pathway in Neuroblastoma

Since OP449 reactivated the phosphatase PP2A, known to inhibit the AKT pathway, and because AKT is important for the growth of NB, we first determined the impact of OP449 on AKT phospho-signaling in SH-SY5Y and KELLY cells. OP449 decreased activating phosphorylation of S473 and T308 in AKT and of p70S6K (**Figure 6B**). Thus, OP449 inhibits activating phospho-signaling of the AKT pathway in NB.

Since OP449 synergized with the kinase inhibitor dasatinib, we investigated their combined effect on the AKT pathway. Dasatinib reduced activating phosphorylations of AKT and p70S6K (**Figure 6B**). Of note, activating phosphorylations of AKT were more reduced by the combination of OP449 with dasatinib compared to single drug use (**Figure 6C**). No synergy was observed in ERK1/2 phosphorylation (**Figure 6C**). In summary, OP449 and dasatinib synergize in inhibiting activating phosphorylations in the AKT pathway of NB cells.

### OP449 Synergizes With Dasatinib in Additional *MYCN*-Amplified and Non-Amplified Neuroblastoma Cells

**Figure 7:**
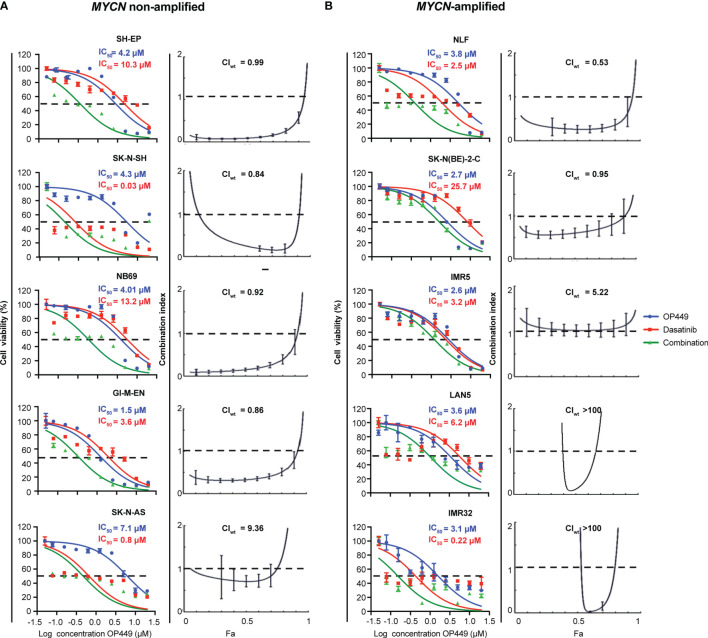
Synergistic effect of OP449 and dasatinib in a panel of *MYCN*-amplified and non-amplified neuroblastoma (NB) cell lines. Cells were treated for 72 h with OP449, dasatinib, or a combination of both. Cell viability determined by MTT is depicted as percentage of untreated cells, and IC_50_ is shown. CI and weighted CI (CI_wt_) were determined. A CI or weighted CI of less than 1 indicates synergy, 1 an additive effect, and greater than 1 an antagonist effect. **(A)** OP449 and dasatinib synergistically inhibit SH-EP, SK-N-SH, NB69, and GI-M-EN but not SK-N-AS *MYCN* non-amplified cells. The left panels show dose-response curves plotted as log of OP449 concentrations ranging from 0 to 20 µM at a molar ratio of 1 (OP449) to 1.25 (dasatinib) for SH-EP cells and 1 to 2.5 for the other cell lines. The right panels show Fa-CI plots. CI_wt_ is stated for each Fa-CI plot. **(B)** OP449 and dasatinib synergistically inhibit NLF and SK-N-BE(2)-C but not IMR5, LAN5, and IMR32 NB *MYCN-*amplified cells. The left panels show dose-response curves plotted as log of OP449 concentrations ranging from 0 to 20 µM at a molar ratio of 1 (OP449) to 2.5 (dasatinib). The right panels show Fa-CI plots. CI_wt_ is stated for each Fa-CI plot. The vertical bars on Fa-CI plots represent 95% confidence intervals. All experiments were repeated three times.

Given that OP449 synergized with dasatinib in SH-SY5Y and KELLY cells, we wanted to know whether OP449 and dasatinib synergize in a broader panel of *MYCN* non-amplified and *MYCN*-amplified NB cell lines. OP449 and dasatinib reduced cell viability at 72 h with IC_50_ ranging at 1.2–7.1 and 0.03–25.7 µM, respectively (**Figure 7**). The CI_wt_ values indicate that OP449 and dasatinib synergistically reduced the viability of SH-EP, SK-N-SH, NB69, and GI-M-EN but not of SK-N-AS *MYCN* non-amplified NB cells (**Figure 7A**) and synergized in NLF and SK-N-BE(2)-C but not in IMR5, LAN5, and IMR32 *MYCN*-amplified NB cells (**Figure 7B**). Together, these data show that OP449 synergizes with dasatinib in many *MYCN*-amplified and non-amplified NB cell lines.

### OP449 Inhibits Neuroblastoma Cells in Part by Inhibiting AKT

**Figure 8:**
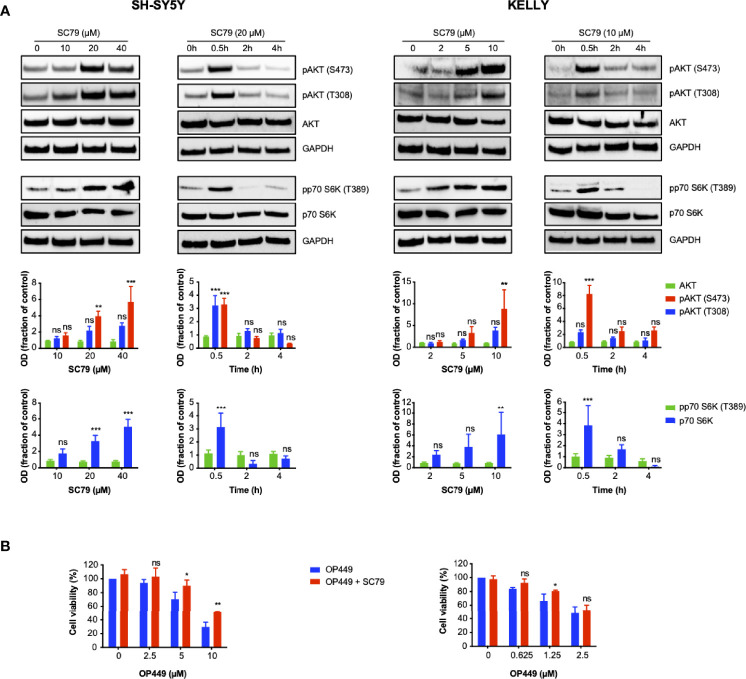
OP449-induced decrease of viability of neuroblastoma (NB) cells is partially rescued by activating AKT. **(A)** AKT is activated by SC79 depending on dose and time. Cells were treated with increasing doses of SC79 for 0.5 h or with fixed doses of 10 (KELLY cells) and 20 µM (SH-SY5Y cells) for increasing time. Cell lysates were subjected to Western blotting. GAPDH was used as loading control. The top panels show representative Western blotting; full-length blotting is depicted in **Supplementary Figures 9, 10**. The bottom panels show means ± SEM of optical density (OD) measurements of Western blotting of 3 independent experiments. Results are expressed as fraction of OD of untreated control cells. Statistical analysis is performed by two-way ANOVA test. **p < 0.01, ***p < 0.001; ns, not significant. **(B)** Viability is partially rescued by AKT activation. Cells were pretreated for 0.5 h with SC79 at 10 µM for KELLY cells and 20 µM for SH-SY5Y cells. SC79 was continued for additional 2 h in the presence of increasing doses of OP449. Results are expressed as percentage of viability of untreated control cells. Statistical analysis is performed by two-way ANOVA test. *p < 0.05, **p < 0.01, ns; not significant.

Given that OP449 inhibited activating phospho-signaling of AKT and that OP449 and dasatinib synergized in this process, we asked whether cellular inhibition by OP449 could be rescued by activating AKT. SC79, an activator of AKT, was used, and activation was determined by phosphorylation of AKT and the downstream p70S6 kinase. For SH-SY5Y and KELLY cells, 20 and 10 µM of SC79 were found to be suitable doses, respectively (**Figure 8A**). AKT activation occurred at 0.5 h and subsided at 2 h (**Figure 8A**). Therefore, these doses and a duration of 2 h were chosen for subsequent rescue experiments. As sensitivity for OP449 was higher in KELLY than in SY5Y cells, lower doses of OP449 were used in KELLY cells. Of note, at higher OP449 concentrations, activation of AKT led to significant, albeit partial rescue, of OP449-mediated viability inhibition, more pronounced in KELLY cells (**Figure 8B**). Thus, inhibition of NB cells by OP449 is in part mediated by AKT inhibition.

## Discussion

This work establishes that PPP2CA is a novel therapeutic target in NB that can be disinhibited by the SET inhibitor OP449, in part mediated by AKT inhibition. When combining OP449-induced reactivation of PP2A with inhibition of kinases, synergy ensues in NB cells.

Reduction of PPP2CA by knock-down decreased growth of NB cells, and complete ablation of PPP2CA by knock-out was not tolerated by the cells. Thus, NB cells are addicted to PPP2CA. However, activation of PPP2CA by the SET inhibitor OP449 also led to growth inhibition of the cells, in line with a tumor suppressor function of PPP2CA. These opposing functions of PPP2CA may appear paradoxical at first sight. However, such a phenomenon, dubbed the “Goldilocks phenomenon,” is also operational in other genes or pathways, such as NOTCH (42), FOXO1 (43, 44), and FOXO3A (45) or the PTEN-PI3K-AKT axis in lymphoid malignancies (46). It describes a bell-shaped cellular response to a tightly regulated protein, with cell death occurring on both sides (“too little” or “too much”) of the bell’s apex (“just right”).

Interestingly, activation of MYCN enhanced control of cell growth by decreased PPP2CA. This may appear counterintuitive, given that *MYCN* is an oncogene conferring poor prognosis to NB when amplified. However, this is in line with MYCN and MYC causing cell death in stressed NB cells (37, 47, 48). Signaling perturbances caused by decreased PP2A activity may constitute such stress.

These results imply that both inhibiting PPP2CA and thus PP2A and enhancing PP2A can control NB cells. However, given that PPP2CA may also be essential for normal cells, enhancing PP2A activity, such as disinhibiting PP2A by inhibiting SET, appears to be a more prudent therapeutic approach. SET mRNA and protein expression was strongly associated with aggressive disease and poor prognosis in patients with NB, suggesting SET as a promising novel target in NB. Indeed, the SET inhibitor OP449 effectively decreased the aggressiveness of KMB cells. This occurred independent of the molecular alterations present in the NB cell lines, including *MYCN* amplification, and was mediated by reactivation of PP2A activity associated with activation of PPP2CA. In line with its function as a serine/threonine-protein phosphatase, reactivated PP2A decreased activating phosphorylation of serine/threonine residues in the AKT pathway. This pathway is crucial for maintaining the aggressiveness of NB cells. As PP2A is one of the most important phosphatases in cells, additional phospho-signaling pathways not investigated in this study may have been affected by OP449.

Several kinase inhibitors are in clinical trials for NB (49–53) including dasatinib (54). Investigating a panel of diverse kinase inhibitors, several were found to synergize with OP449. Prominent among them was dasatinib. While dasatinib may inhibit the tyrosine kinase Src in NB (55) and thus downstream AKT, other pathways acting on AKT might also be targeted by dasatinib. Synergistic cell killing by OP449 with dasatinib was associated with a more pronounced decrease of activating AKT phosphorylations. This suggests enhanced activation of the AKT pathway as one of the molecular mechanisms of synergy between OP449 and dasatinib. Additional phospho-signaling pathways not assessed may also play a role in this synergy. Differential activity of these pathways may explain why some cell lines have not responded to the combination.

There are limitations to this study. First, future investigations are warranted to assess the efficacy of OP449 against NB *in vivo.* Along this line, OP449 has been shown to decrease the growth of other cancer xenografts in mice (24, 26–28, 35, 36, 56). Typically, a dose of 5 mg/kg was given systemically several times per week for up to 18 days. Importantly, the effective OP449 concentrations used for *in vitro* investigations in these studies ranged from 1 to 5 µM. As these correspond to the OP449 concentrations used in our study, concentrations of OP449 effective against NB should be achievable *in vivo*. Second, while the aforementioned studies did not reveal toxicity in adult mice, juvenile toxicity studies in young mice are required before the use of OP449 in children with NB can be considered. Third, while induction of AKT rescued the inhibition of NB cells by OP449, the rescue was partial. Thus, additional molecular mechanisms mediating the cellular effects of OP449 must be operational. These may also involve mechanisms that are independent of PP2A.

In summary, reactivation of PP2A by OP449 and combining OP449 with kinase inhibitors hold promise for the therapy of NB, warranting further investigations.

## Data Availability Statement

The datasets presented in this study can be found in online repositories. The names of the repository/repositories and accession number(s) can be found in the article/**Supplementary Material**.

## Author Contributions

CB and CG conceived and designed the study. CG and MD performed and analyzed the experiments. MV provided the reagents. KMD contributed to the interpretation of the results. CG and CB wrote the manuscript. All authors read and approved the submitted version of the manuscript.

## Funding

This work was supported by grant 2018_A27 of the Else Kröner-Fresenius Stiftung (to CG).

## Conflict of Interest

Author MV was employed by Cognosci, Inc.

The remaining authors declare that the research was conducted in the absence of any commercial or financial relationships that could be construed as a potential conflict of interest.

## Publisher’s Note

All claims expressed in this article are solely those of the authors and do not necessarily represent those of their affiliated organizations, or those of the publisher, the editors and the reviewers. Any product that may be evaluated in this article, or claim that may be made by its manufacturer, is not guaranteed or endorsed by the publisher.
