# Supplementary material for: PPP2CA Is a Novel Therapeutic Target in Neuroblastoma Cells That Can Be Activated by the SET Inhibitor OP449
Source: Front Oncol. 2022 Jun 22;12:744984. doi: 10.3389/fonc.2022.744984 (PMC9258974; doi:10.3389/fonc.2022.744984)
Supplement: Supplementary file 1 [file DataSheet_1.pdf]

Supplementary Material

Supplementary Data

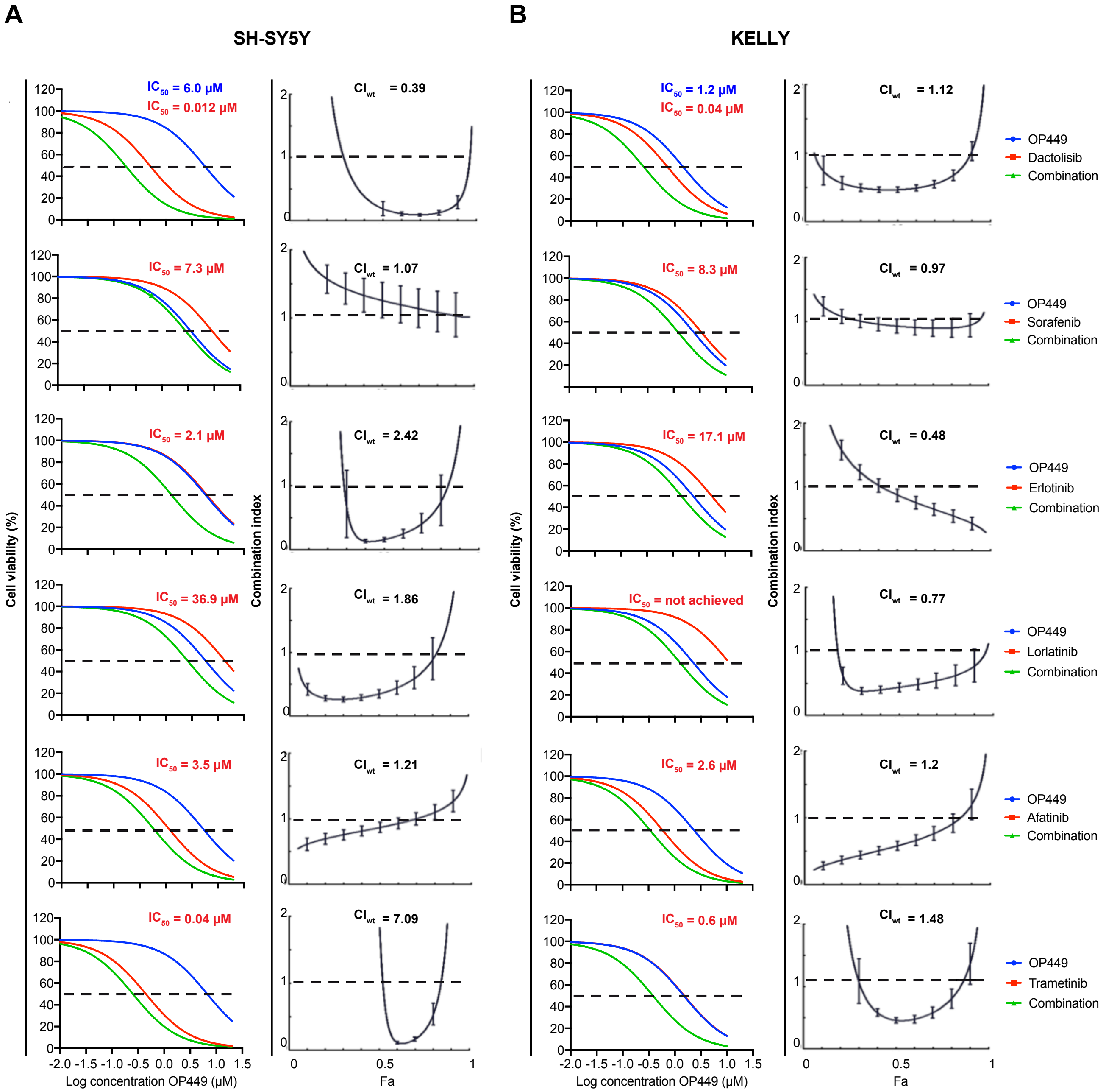

Suppl. Fig. 1 Synergy of OP449 and kinase inhibitors in SH-SY5Y and KELLY NB cells. Cells were treated for 72 h with OP449, a kinase inhibitor or a combination of both. Cell viability was determined by MTT and is depicted as percentage of untreated cells, with IC_{50} values being shown. Combination Indices (CI) and weighted average Combination Indices (CI_{wt}) were determined according to the Chou-Talalay method. A CI or CI_{wt} less than 1 indicates synergy, of 1 an additive effect and greater than 1 an antagonist effect. Two independent experiments were performed with similar results. **(A) Synergy in SH-SY5Y cells.** The left panels show dose response curves plotted as log of OP449 concentrations ranging from 0 to 20 µM at a molar ratio of 1 (OP449) to 4 (kinase inhibitors). The right panels show Fa (Fraction affected) - CI plots. CI_{wt} is stated for each Fa-CI plot. The vertical bars on Fa-CI plots represent 95% confidence intervals. **(B) Synergy in KELLY cells.** The left panels show dose response curves plotted as log of OP449 concentrations ranging from 0 to 20 µM at a molar ratio of 1 (OP449) to 2.5 (kinase inhibitors). The right panels show Fa-CI plots. CI_{wt} is stated for each Fa-CI plot.

A

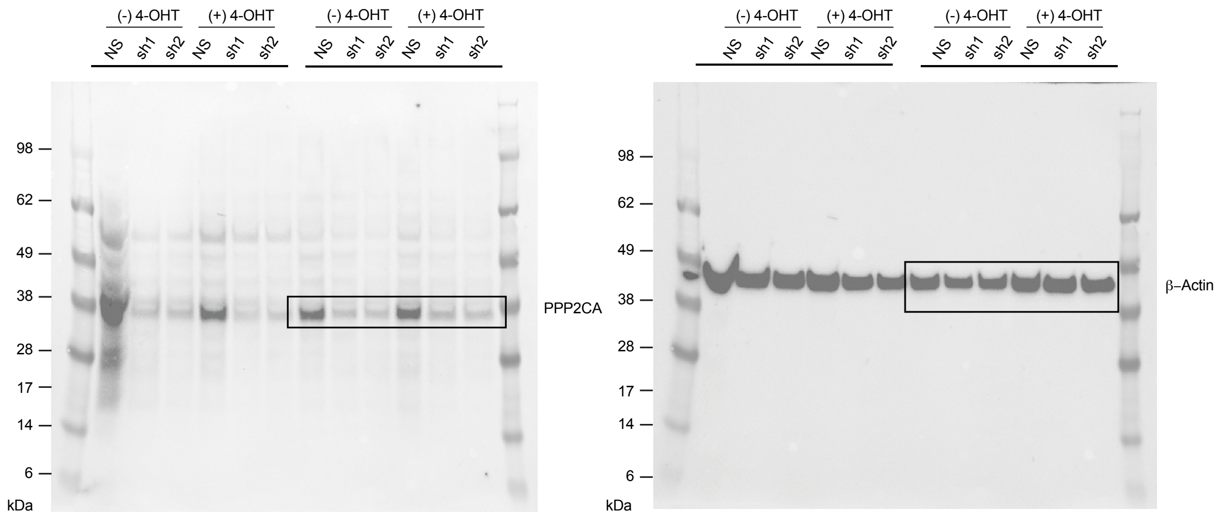

B

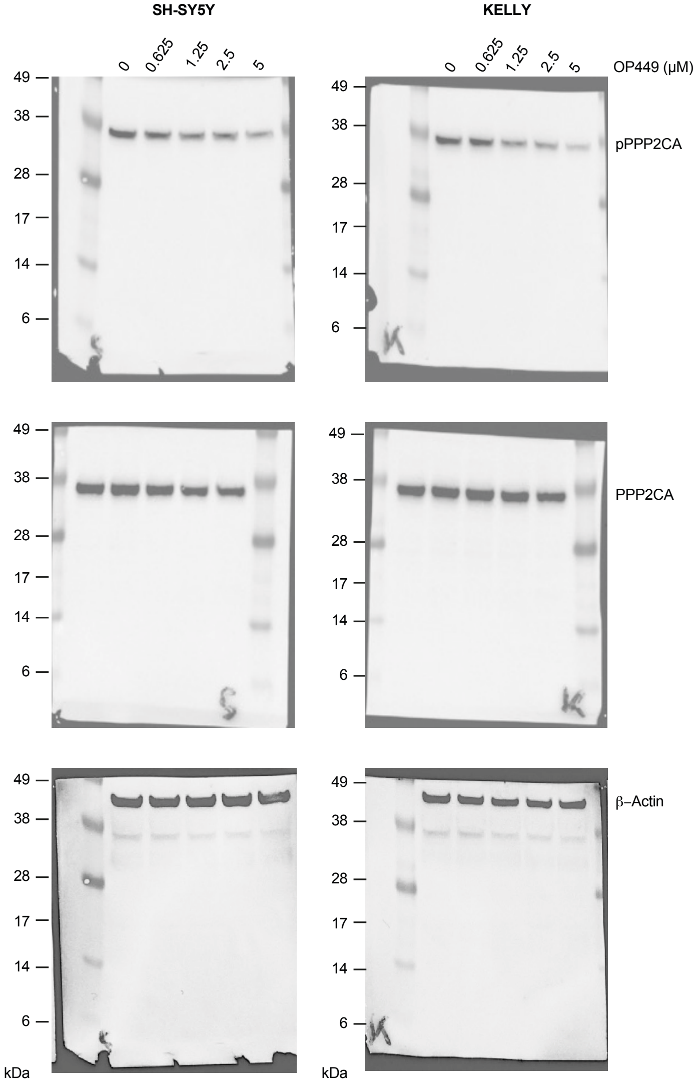

Suppl. Fig. 2 Uncropped full-length Western blots of Fig. 2A (A) and Fig. 5A (B).

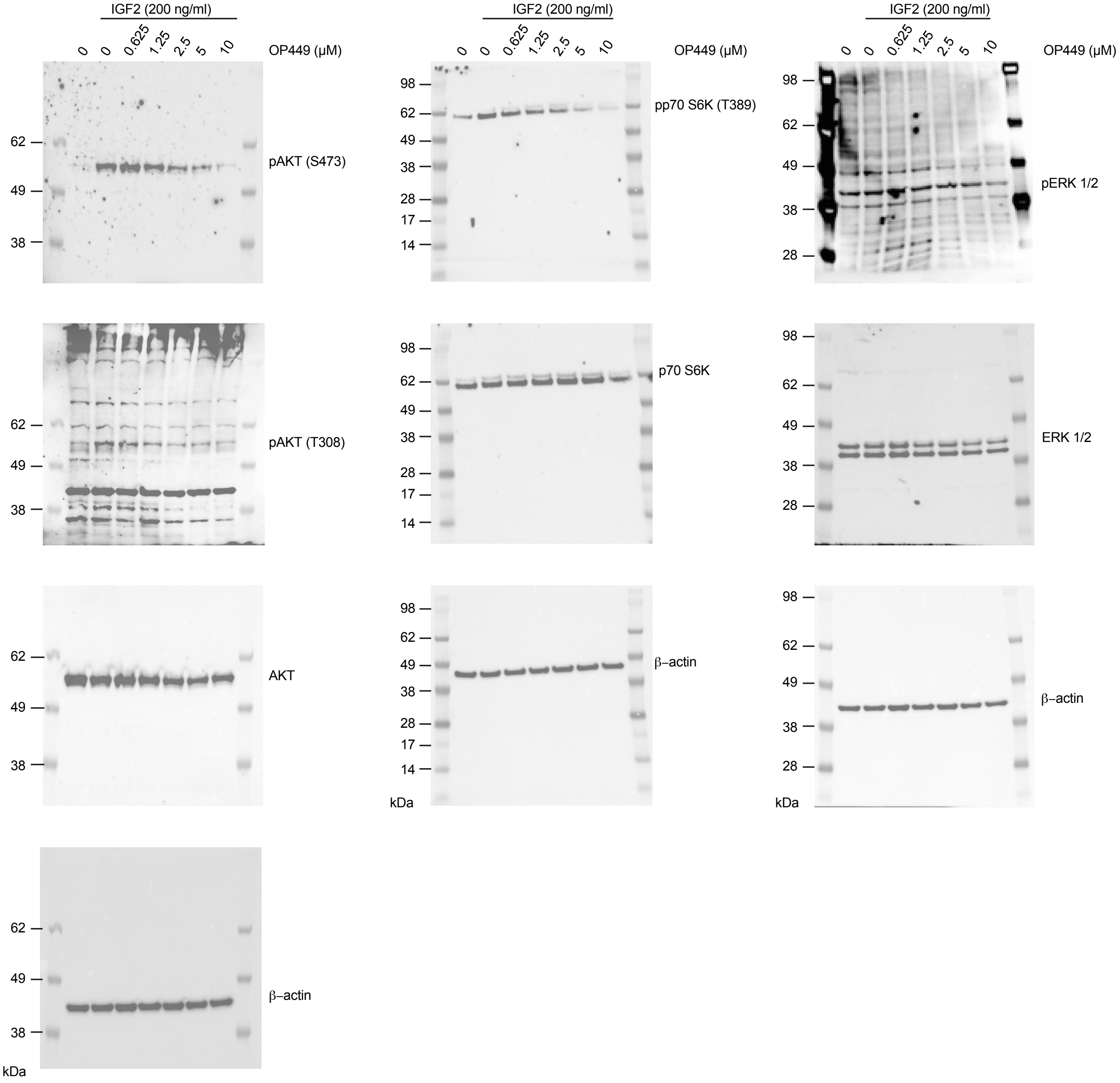

Suppl. Fig. 3 Uncropped full-length Western blots Fig. 6B left upper panel.

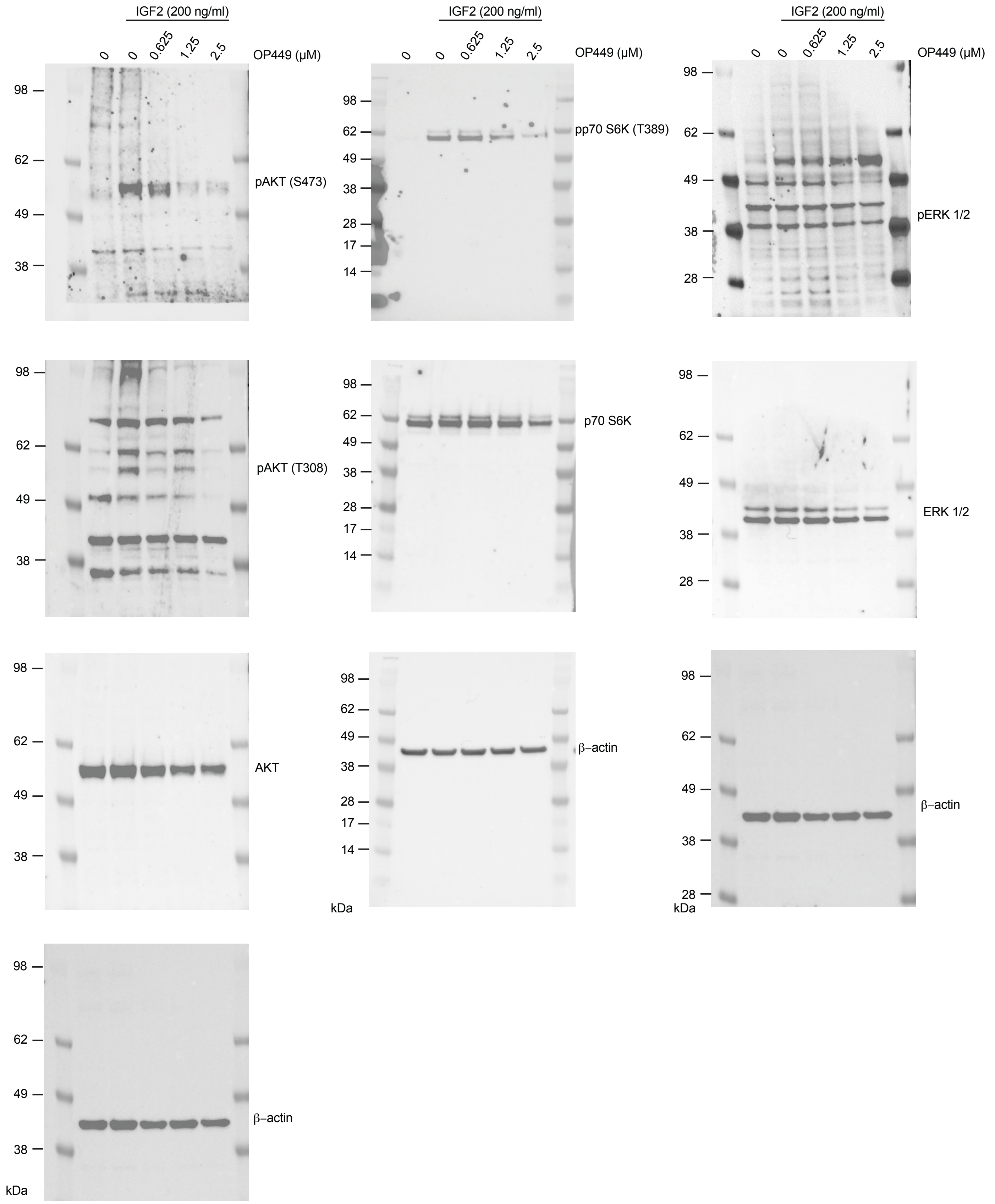

Suppl. Fig. 4 Uncropped full-length Western blot Fig. 6B right upper panel.

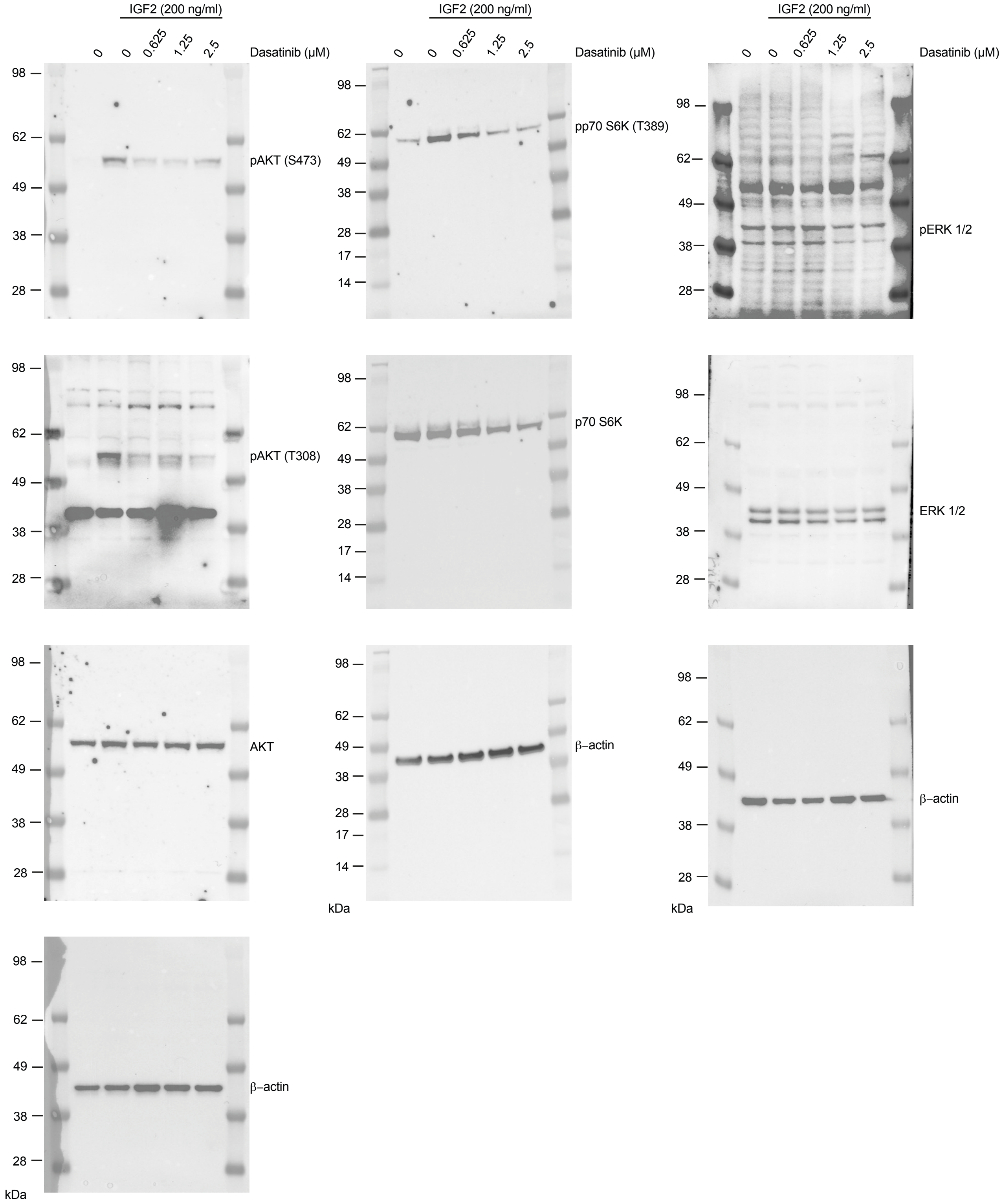

Suppl. Fig. 5 Uncropped full-length Western blots Fig. 6B left lower panel.

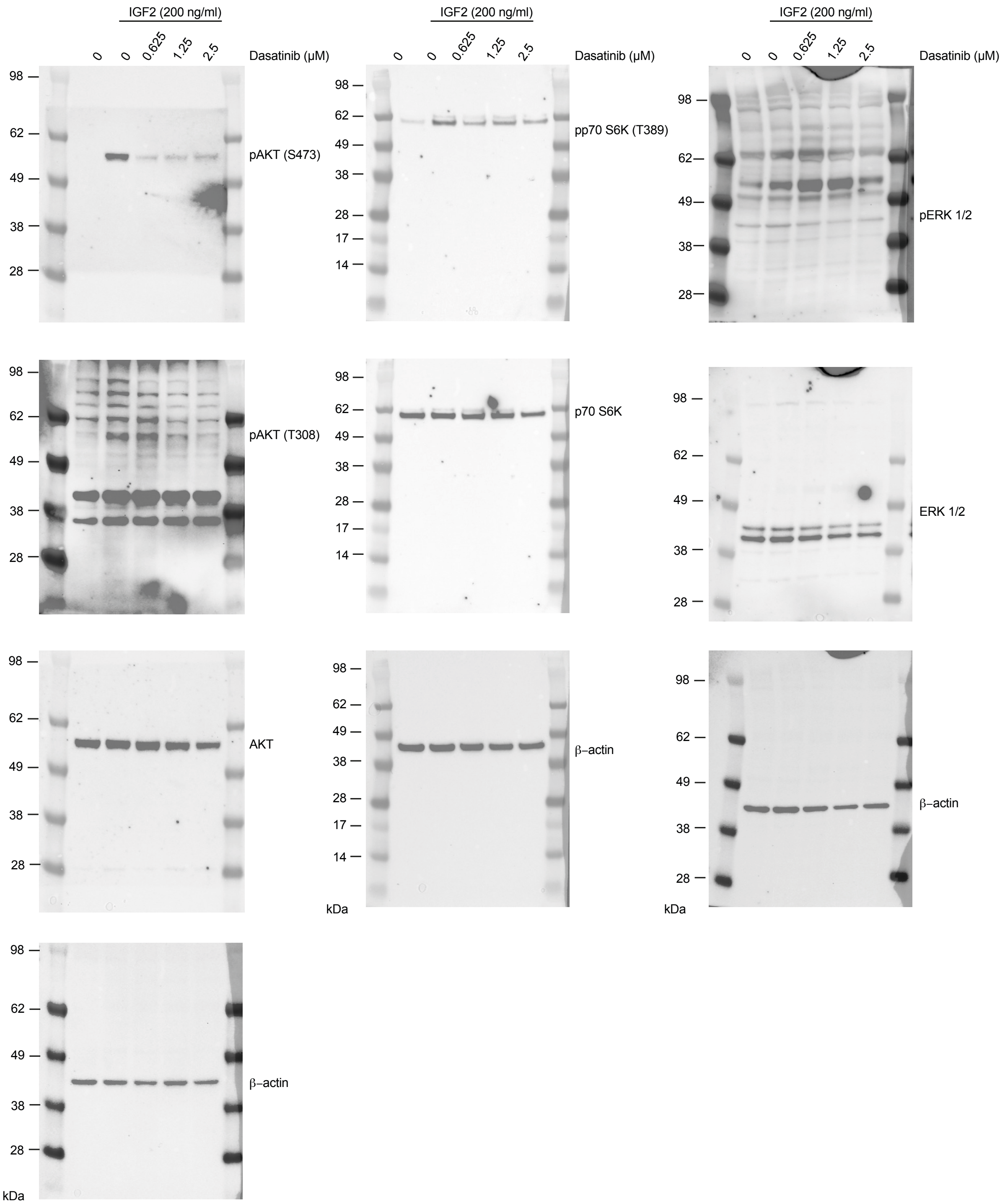

Suppl. Fig. 6 Uncropped full-length Western blot Fig. 6B right lower panel.

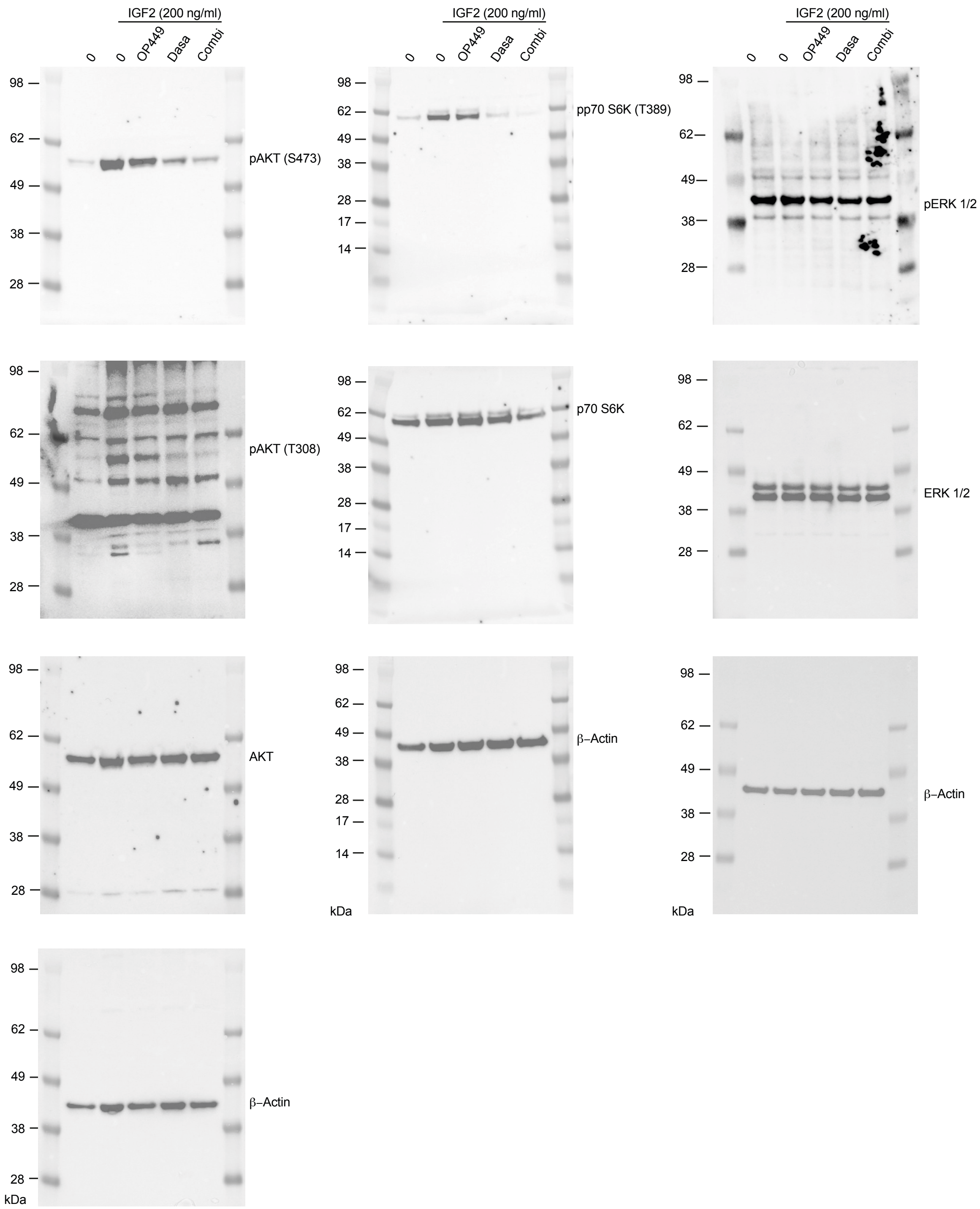

Suppl. Fig. 7 Uncropped full-length Western blots of Fig. 6C left panel.

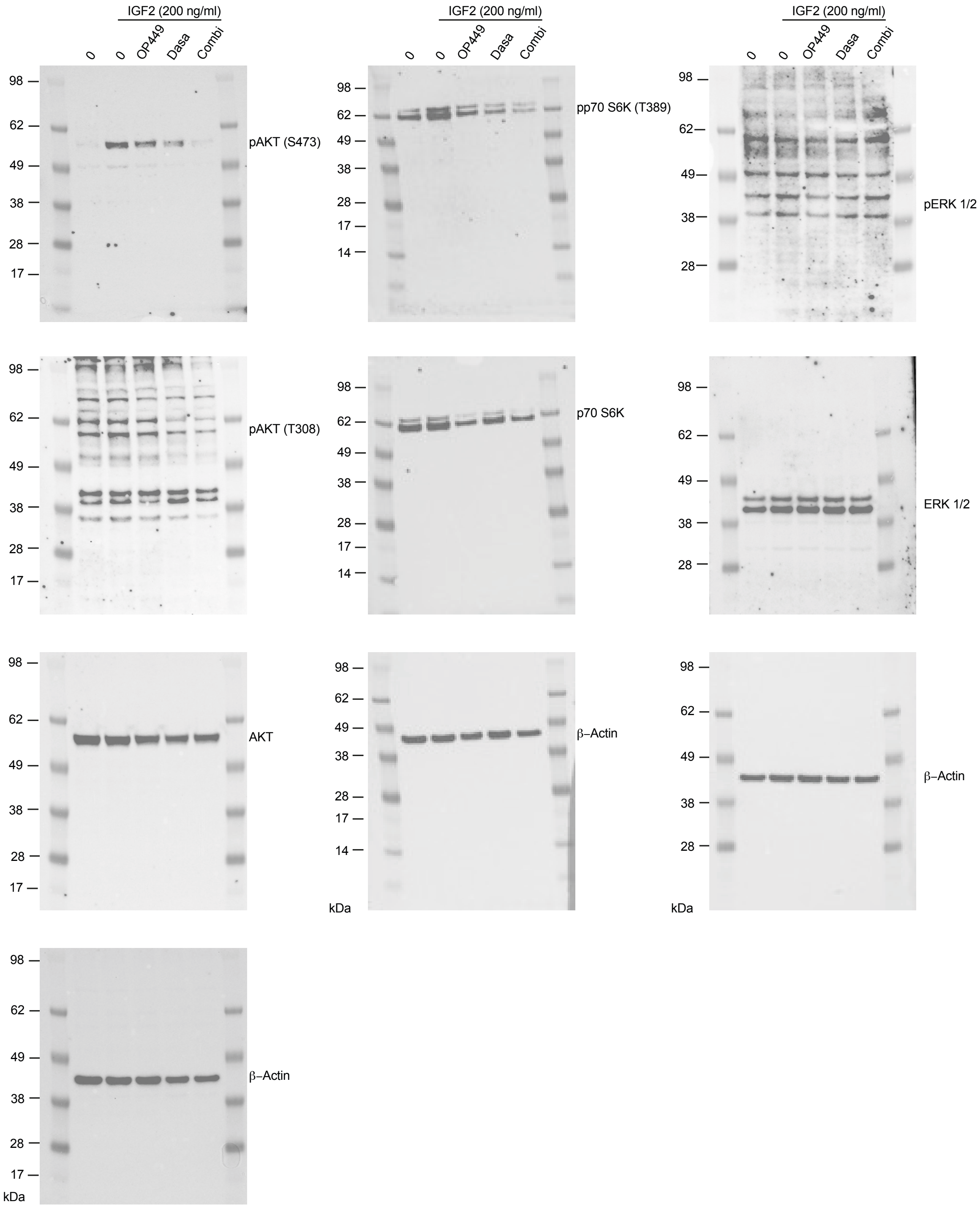

Suppl. Fig. 8 Uncropped full-length Western blots of Fig. 6C right panel.

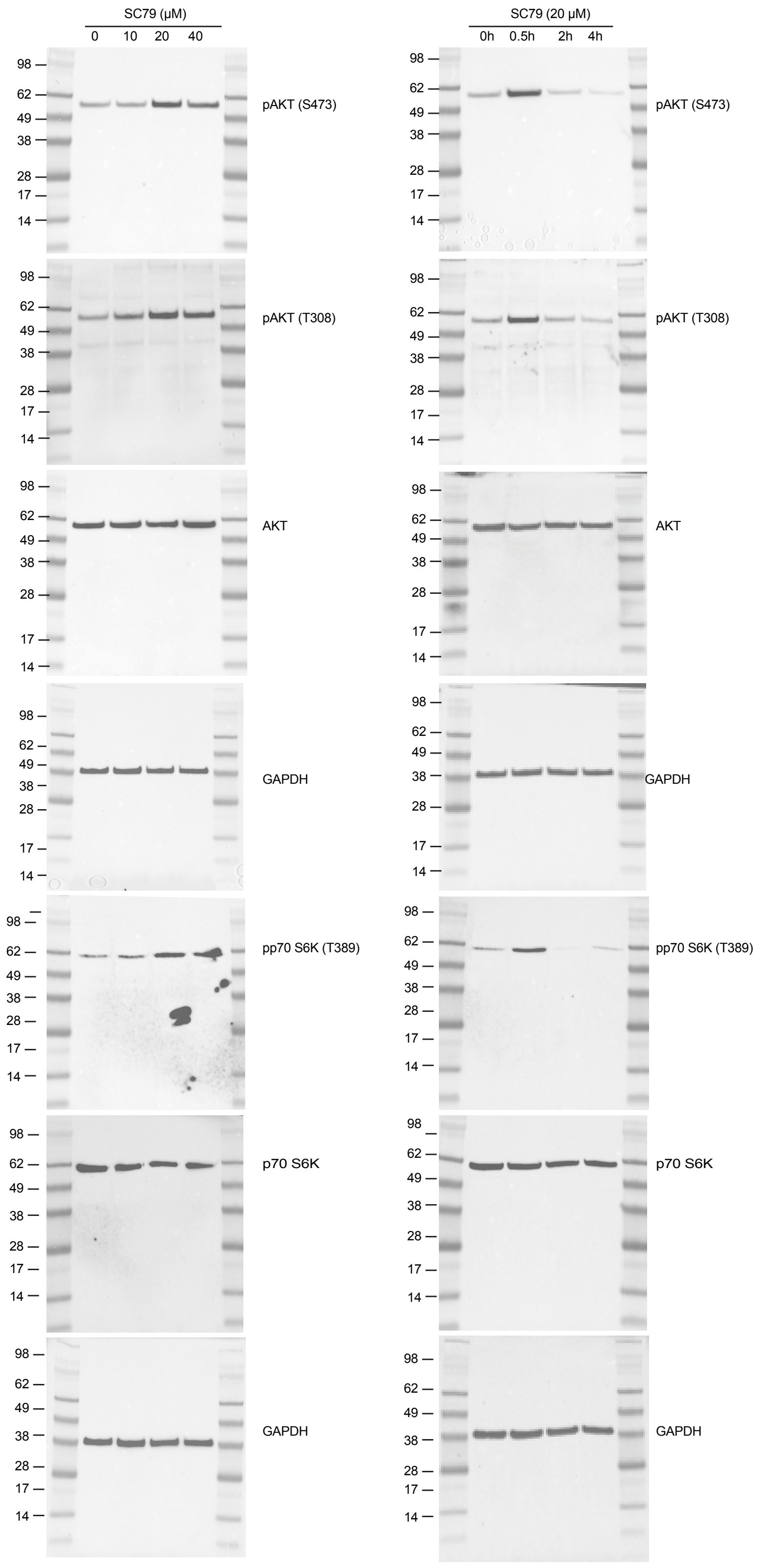

Suppl. Fig. 9 Uncropped full-length Western blots of Fig. 8A left panel.

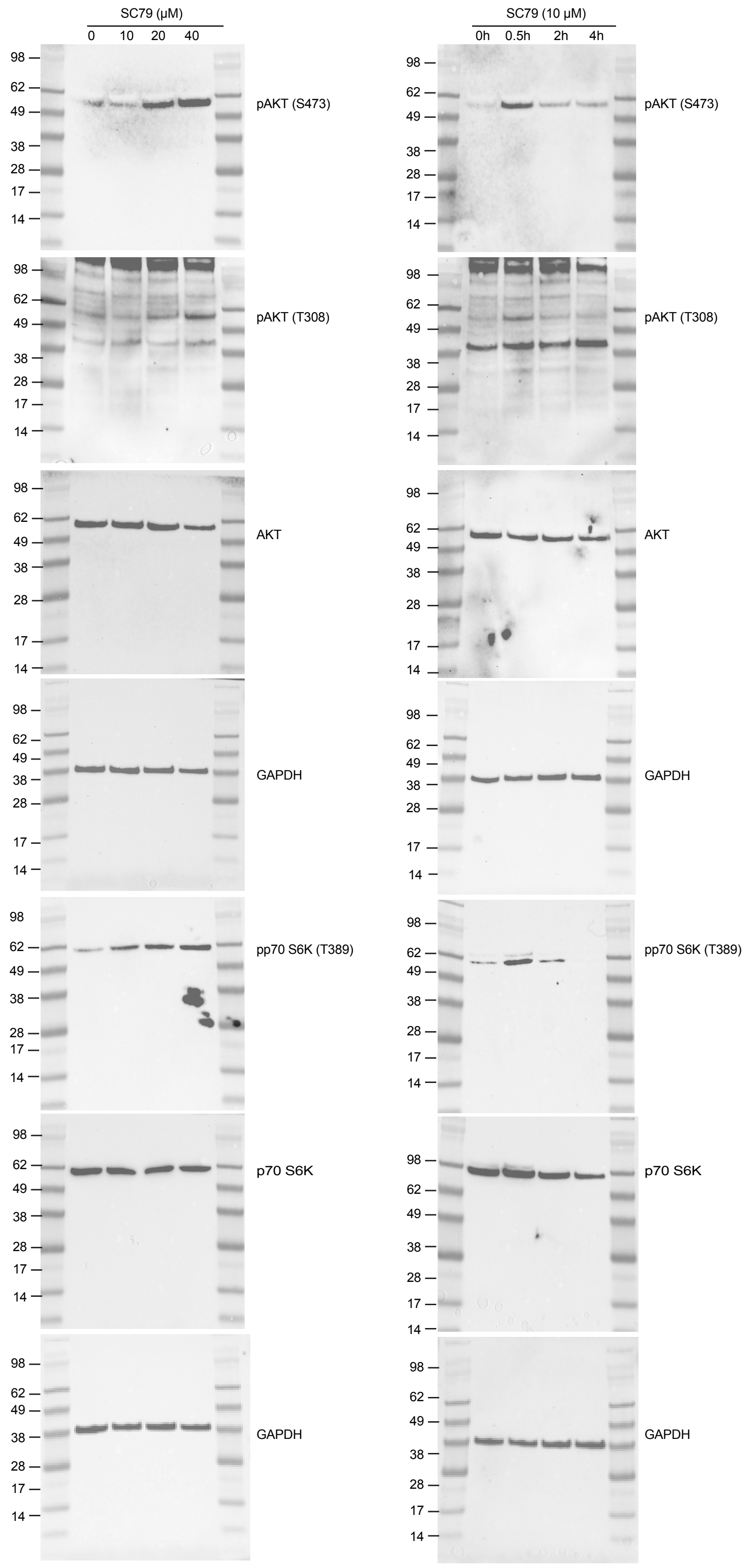

Suppl. Fig. 10 Uncropped full-length Western blots of Fig. 8A right panel.
